# Selecting optimal second-generation antihistamines for allergic rhinitis and urticaria in Asia

**DOI:** 10.1186/s12948-017-0074-3

**Published:** 2017-11-01

**Authors:** Marysia Tiongco Recto, Ma. Teresita Gabriel, Kanokvalai Kulthanan, Pongsakorn Tantilipikorn, Derrick Chen-Wee Aw, Tak Hong Lee, Ch’ng Chin Chwen, Somasundran Mutusamy, Nguyen Trong Hao, Vo Thanh Quang, Giorgio Walter Canonica

**Affiliations:** 1 University of the Philippines-Philippine General Hospital, Manila, Philippines; 2 Research Institute for Tropical Medicine, Manila, Philippines; 3 Faculty of Medicine Siriraj Hospital, Mahidol University, Bangkok, Thailand; 4 Department of General Medicine, Sengkang Health, Singapore, Singapore; 5 Allergy Centre, Hong Kong Sanatorium and Hospital, Hong Kong, Hong Kong; 6 University Malaya Medical Centre, Kuala Lumpur, Malaysia; 7 Hospital Kuala Lumpur, Kuala Lumpur, Malaysia; 8 Ho Chi Minh City Hospital of Dermatology and Venereology, Ho Chi Minh City, Vietnam; 9 National Hospital of Otorhinolaryngology, Hanoi, Vietnam; 10 Personalised Medicine Asthma & Allergy Clinic, Humanitas University, Clinical & Research Hospital, Rozzano, Milan, Italy

**Keywords:** Allergic rhinitis, Antihistamines, Bilastine, Treatment algorithm, Urticaria

## Abstract

**Background:**

Allergic diseases are on the rise in many parts of the world, including the Asia–Pacific (APAC) region. Second-generation antihistamines are the first-line treatment option in the management of allergic rhinitis and urticaria. International guidelines describe the management of these conditions; however, clinicians perceive the additional need to tailor treatment according to patient profiles. This study serves as a consensus of experts from several countries in APAC (Hong Kong, Malaysia, the Philippines, Singapore, Thailand, Vietnam), which aims to describe the unmet needs, practical considerations, challenges, and key decision factors when determining optimal second-generation antihistamines for patients with allergic rhinitis and/or urticaria.

**Methods:**

Specialists from allergology, dermatology, and otorhinolaryngology were surveyed on practical considerations and key decision points when treating patients with allergic rhinitis and/or urticaria.

**Results:**

Clinicians felt the need for additional tools for diagnosis of these diseases and a single drug with all preferred features of an antihistamine. Challenges in treatment include lack of clinician and patient awareness and compliance, financial constraints, and treatment for special patient populations such as those with concomitant disease. Selection of optimal second-generation antihistamines depends on many factors, particularly drug safety and efficacy, impact on psychomotor abilities, and sedation. Country-specific considerations include drug availability and cost-effectiveness. Survey results reveal bilastine as a preferred choice due to its high efficacy and safety, suitability for special patient populations, and the lack of sedative effects.

**Conclusions:**

Compliance to the international guidelines is present among allergists, dermatologists and otorhinolaryngologists; however, this is lower amongst general practitioners (GPs). To increase awareness, allergy education programs targeted at GPs and patients may be beneficial. Updates to the existing international guidelines are suggested in APAC to reflect appropriate management for different patient profiles and varying symptoms of allergic rhinitis and urticaria.

**Electronic supplementary material:**

The online version of this article (doi:10.1186/s12948-017-0074-3) contains supplementary material, which is available to authorized users.

## Background

Allergic diseases comprise a variety of conditions including allergic rhinitis, allergic asthma, atopic dermatitis, contact allergies, and food allergies. Urticaria can also occur either as an allergic or nonallergic response. The global prevalence of allergic diseases is up to 30% [1]. Statistics on the prevalence of allergic diseases in APAC are scarce; however, the Allergies in Asia–Pacific Survey demonstrated that the prevalence of allergic rhinitis ranged from 2.5 to 44.2% [2, 3]. Due to rapid economic development and urbanization in Asia, the prevalence of allergic diseases in this region is expected to rise over the next two decades [4].

Allergic rhinitis is classified as “intermittent” (symptoms for < 4 days a week), “persistent” (symptoms present for > 4 days a week and for more than 4 consecutive weeks), “mild”, and “moderate” according to the Allergic Rhinitis and its Impact on Asthma (ARIA) guidelines [5]. Meanwhile, urticaria is classified as “spontaneous” (acute spontaneous [spontaneous wheals < 6 weeks] or chronic spontaneous [spontaneous wheals > 6 weeks]), and “physical” according to European Academy of Allergology and Clinical Immunology/Global Allergy and Asthma European Network/European Dermatology Forum/World Allergy Organization (EAACI/GA(2)LEN/EDF/WAO) guidelines [6].

Allergic diseases pose a huge socioeconomic impact in terms of loss of productivity and effect on the patient’s quality of life. In the European Union, an allergic person was estimated to have symptoms for an average of 51 working days per annum, hence affecting work performance and leading to absenteeism [7]. The duration of symptoms is expected to be higher in APAC due to the presence of perennial allergens. For example, the prevalence of perennial allergic rhinitis in APAC ranges from 34 to 41% in comparison to 15% in non-APAC countries [2, 8]. A patient with persistent allergic rhinitis could be suffering throughout the year where symptoms could be mild to severe, thereby impairing daily activities. A study in APAC showed that patients with allergic rhinitis reported a significant amount of discomfort during nasal allergy attacks, including nasal obstruction, with 42% of patients lacking a good night’s sleep and 38% reporting moderate-to-severe impact on daily life [2]. Patients with chronic urticaria also reported reductions in the Satisfaction Profile score for the amount and quality of their sleep, physical wellbeing, resistance to stress, and mood [9].

H1 antihistamines interfere with histamine action and downregulate allergic inflammation [10]. They are the first-line treatment options for both persistent allergic rhinitis and chronic urticaria, as recommended by the ARIA and EAACI/GA(2)LEN/EDF/WAO guidelines [5, 6]. Second-generation antihistamines are recommended over the first-generation antihistamines, due to their favorable efficacy/safety ratio, pharmacokinetics, and lack of anticholinergic and sedative side effects [5, 6, 11]. Before prescribing pharmacotherapy, factors for consideration include efficacy, safety, cost-effectiveness, patient preference, goals of treatment, anticipated adherence to treatment, disease severity and control, and presence of concurrent conditions [5].

The ARIA and EAACI/GA(2)LEN/EDF/WAO guidelines are used in some APAC countries where country-specific guidelines do not exist [12, 13]. A survey done in Malaysia showed high satisfaction with the recommendations from the current ARIA guidelines; between 58 and 89% of Ear, Nose, and Throat (ENT) specialists, pharmacists, and GPs [13]. However, it was observed in a World Allergy Organization German study that only 14.8% of 121,593 patients with allergic rhinitis were treated according to the ARIA recommendations; there are no data to suggest a difference in compliance in Asian countries. In this study, 36.1% of patients who were treated by ENT specialists received therapy according to guidelines, while only 16% of GPs heeded the recommendations [14]. In the management of chronic urticaria, in agreement with the EAACI/GA(2)LEN/EDF/WAO guidelines, the Asian Academy of Dermatology and Venereology Study Group arrived at a consensus that second-generation non-sedating antihistamines should be prescribed as the first-line treatment [6, 12].

However, in the case of chronic spontaneous urticaria in an Irish cohort, only 11% of those who were treated by GPs were managed with non-sedating antihistamines, a recommendation derived from the EAACI/GA(2)LEN/EDF/WAO guidelines; this increased to 28% following specialist review [15]. The above suggests that there remains an unmet need with regard to bringing the optimal treatment options to patients for the management of allergic rhinitis and urticaria in APAC. Many patients experience sedation from the usage of first-generation antihistamines [16]. In addition, the lack of persistent relief of symptoms, and the need for better therapies with fewer side effects, call for improvements or updates to the current treatment guidelines [17].

The objective of this article is to discuss current guidelines in the context of APAC considerations, i.e., compliance and practical application, and to propose updates to the current treatment algorithms in the selection of second-generation antihistamines to streamline treatment decision-making for the management of allergic rhinitis and urticaria. This would be done in view of the most common patient profiles found within APAC, bearing in mind the challenges and limitations when implementing existing guidelines in the region. Ways to ensure that patients receive the best possible standard of care are also discussed. Specifically, this manuscript will provide guidance on the selection of the optimal antihistamines for the appropriate patients based on clinical evidence and existing treatment algorithms used by clinicians, to provide a convenient tool for specialists and GPs.

## Methods

A consensus of ten experts from APAC, comprising dermatologists, ENT specialists, and allergy specialists (n = 5, n = 3, and n = 2, respectively) practicing in Hong Kong, Malaysia, the Philippines, Singapore, Thailand, and Vietnam, was obtained through a written survey (see Additional file 1). Information was collected from the experts on the awareness of, and compliance with, the ARIA and EAACI/GA(2)LEN/EDF/WAO guidelines and clinical practices of their peers and GPs.

Clinicians were asked to describe common patient profiles in their clinical practice to understand the practical considerations and/or limitations that determine the preferred method of diagnosis and treatment. Additionally, clinicians’ views, preferences, and considerations for the choice of treatment for allergic rhinitis and urticaria using second-generation antihistamines were surveyed. The findings from the survey were then used to generate an optimal treatment algorithm for common patient profiles encountered in their clinical practice.

## Results

### Awareness of, and compliance with, ARIA and EAACI/GA(2)LEN/EDF/WAO guidelines

Survey results revealed a high awareness of the ARIA and EAACI/GA(2)LEN/EDF/WAO guidelines. Eighty per cent of ENT and allergy specialists generally follow ARIA guidelines but 20% tailor treatment according to their patients. It was highlighted that current ARIA guidelines, however, lack recommendations for severe patient groups such as those with Samter’s triad, and clinicians felt the need for these inclusions in the guidelines. It was also mentioned that 40% of ENT and allergy specialists prefer to follow country-specific guidelines such as the ‘Clinical Practice Guidelines of Allergic Rhinitis of Thailand’ and ‘British Society of Allergy and Clinical Immunology Guidelines’. In contrast, all dermatologists and allergists surveyed tend to follow EAACI/GA(2)LEN/EDF/WAO guidelines for urticaria. However, they indicated that there may be instances of noncompliance where other clinicians in the region lack awareness of dosage adjustments, or have individual preferences for the use of antihistamine combinations.

The surveyed clinicians indicate that they perceived GPs would fully, or partially, adhere to the ARIA and EAACI/GA(2)LEN/EDF/WAO guidelines, although a fraction may not be aware of their existence. The lack of national (local) guidelines and the fear of prescribing or using steroids were cited as potential contributors to nonadherence to guidelines. It was also highlighted that some GPs still use first-generation antihistamines, due to cheaper pricing and patient preferences relating to the perceived advantages of their sedative effects at night, despite being discouraged by both the ARIA and EAACI/GA(2)LEN/EDF/WAO guidelines.

### Patient profiles, practical treatment guidelines, and associated challenges or limitations for allergic rhinitis

All ENT and allergy specialists surveyed commonly see persistent or intermittent allergies among their patients. Survey results revealed the most common patient profiles, unmet needs, key decision points for treatment, and practical considerations and/or limitations in treatment of patients with allergic rhinitis (Tables 1, 2). Specifically, the most common patient profiles were “young children” and adults with allergic rhinitis, asthma, and other comorbidities. Some of these patients present with accompanying sinusitis, nasal polyps, asthma, food allergies, and eczema. A fraction of these patients also present with Samter’s triad.

**Table 1:** Considerations, unmet needs and challenges in implementing treatment guidelines for allergic rhinitis and urticaria

	Allergic rhinitis	Urticaria
Common adult patient profiles	Adults, some with comorbidities (e.g., asthma)	20‒40-year-old adults Pregnant/lactating women
Unmet needs	Inadequate diagnosis Inadequate control of symptoms Lack of long-acting, non-sedative drugs (limited availability) Lack of rest (limited quality of sleep)	Requirement of additional tools for diagnosis (lack of guideline education and availability of tests for specific urticaria subtypes)^a^ Inadequate control of symptoms A single drug with all preferred features of an antihistamine Lack of rest (limited quality of sleep)
Challenges in implementation of existing guidelines	Patient awareness Doctor awareness	Institutional practices and drug availability
Practical considerations in treatment	Patient and doctor education Patient profile (e.g., renal/hepatic impairment, age, concomitant disease) Financial constraints Considerations of taking antihistamines by special populations, such as elderly patients and pregnant or breastfeeding women	Lack of patient compliance Considerations of taking antihistamines by special populations, such as elderly patients and pregnant or breastfeeding women Patient profile (e.g., renal/hepatic impairment, age, concomitant disease) Financial constraints
Limitations in treatment	Patient preferences (demand for a treatment that will restore their QoL)	Patient preferences (demand for a treatment that will restore their QoL)

Data presented in this table reflect results from completed surveys and consensual agreement from authors

*QoL* quality of life

^a^Ancillary tests to diagnose autoimmune urticaria (anti-FCeR1 receptor) and other subtypes of urticaria (i.e. cold urticaria) are not available in parts of the Asia Pacific region

**Table 2:** Key decision points for treatment of allergic rhinitis and urticaria

Key decision points for treatment	Allergic rhinitis	Urticaria
Patient profiles and associated symptoms	Age Duration and severity of symptoms Comorbidities Concomitant drug use Family history of atopic diseases	Age Presence and severity of symptoms Comorbidities Concomitant drug use
Key decision points for diagnosis	Results from skin prick test Presence of clinical symptoms Previous experience with antihistamine	Clinical history Trigger factors Previous experience with antihistamines
Key decision points for treatment	Severity Age Comorbidities Pregnancy and breastfeeding	Disease severity Patient preference Comorbidities Pregnancy and breastfeeding
Importance of patient preference	Use of non-sedating antihistamines and non- anticholingergic antihistamines with fast onset of action Affordable antihistamines with no adverse effects and minimal drug-interaction	

Survey results indicate that an inaccessibility to allergy testing due to the lack of allergy specialists and diagnostic tests in many South-East Asian countries, especially in rural areas, led to inadequate diagnosis of allergic diseases. Furthermore, suitable allergen extracts for allergy testing in APAC, such as local grasses or pollen, are lacking and they may differ from those of the Western world. Other unmet needs include the lack of access to immunotherapy and rhinologic examinations for comorbidities such as nasal endoscopy (for the detection of nasal polyps and rhinosinusitis), and the lack of diagnosis of patients with mixed rhinitis and local allergic rhinitis. The need for a long-acting and non-sedative antihistamine, better immunotherapy vaccines, and other biologics (e.g., lysine acetylsalicylate for provocation testing in cases of suspected Samter’s triad) for severe cases, such as patients with nasal polyposis, were also mentioned.

There are several aspects to diagnosis, and therefore treatment decision-making, for patients with allergic rhinitis, including the skin prick test recommended by the ARIA guidelines [5]. Responses from the survey show, however, that the latter may not be consistently applied across clinical practices—some clinicians may only perform it if patients do not respond to specific treatment possibly due to the dearth of allergy specialists in the region. The presence of clinical symptoms (seasonal or due to environmental triggers) and accompanying symptoms (nasal blockage, rhinorrhea, sinusitis, and nasal polyps) also serve as key diagnostic considerations for allergic rhinitis.

Patient profiles such as age, symptoms and severity (intermittent or persistent), presence of hepatic or renal impairment, concomitant disease, pregnancy, and familial history of atopic diseases, such as allergic rhinitis or asthma, influence treatment decisions. For example, EAACI/GA(2)LEN/EDF/WAO guidelines recommend second-generation H1 antihistamines for pregnant patients. The pattern of symptom occurrence is also another consideration—some patients report an inadequate control of bothersome symptoms, while others experience symptoms of allergic rhinitis that occur in the morning and gradually improve by midday. The presence of symptoms often leads to poorer quality of sleep. Treatment is also determined by previous experience with antihistamines and drug allergies (including those to aspirin and nonsteroidal anti-inflammatory drugs). Table 3 describes the criteria for defining responders versus nonresponders to treatment, and controlled versus uncontrolled symptoms, in the context of allergic rhinitis based on visual analog scale scoring or improvement in symptoms and quality of life post-treatment. The survey also highlighted the role of patients in treatment decision-making where they may demand therapies that restore their quality of life, enabling them to perform their daily activities.

**Table 3:** Definitions of “responders” and “non-responders”, “controlled” and “uncontrolled” symptoms for allergic rhinitis and urticaria

	Allergic rhinitis	Urticaria
Responder	Improvement of overall symptoms by VAS. VAS decreases by > 50% compared with 4‒6 weeks before treatment OR > 50% improvement in symptoms after 1 week of treatment	Absence of, or reduction in number and/or frequency of, urticaria lesions/angioedema OR Complete response: UAS7 score decreases by > 90% from the baseline score Significant improvement: UAS7 score decreases by > 30%, but < 90% from the baseline score
Nonresponder	VAS changes < 50% compared with 4‒6 weeks before treatment OR < 20% improvement in symptoms after 1 week of treatment	Same (or increase in) number and/or frequency of urticaria lesions/angioedema after treatment of adequate dosage for at least 2 weeks OR UAS7 score decreases by < 30% from the baseline score
Controlled symptoms	VAS score < 5/10 at the time of asking the patient OR 80% improvement in symptoms and QoL	Improvement or elimination of itch and/or visible lesions and/or QoL (as perceived by patient) OR UAS7 score decreases by > 90% from the baseline score
Uncontrolled symptoms	VAS score ≥ 5/10 at the time of asking the patient OR < 50% improvement in symptoms and QoL	No change (or worsening) of itch and/or visible lesions and/or quality of life (as perceived by the patient) OR UAS7 score decreases by < 30% from the baseline score or flares up

*QoL* quality of life, *UAS7* weekly urticaria activity score, *VAS* visual analog scale

Clinicians surveyed indicate that patients with lower compliance may be preferentially given ‘once-daily’ drugs. Patient preference and lifestyle could also impact compliance; those with an active lifestyle may prefer non-sedative once-daily pills with a rapid onset of action, while a minority prefer slightly sedating antihistamines at night.

The results underscored several limitations to the treatment of allergic rhinitis, including financial constraints and misdiagnosis by non-medical professionals—parents of young patients often mistake their upper respiratory tract infections for allergic rhinitis. Other limitations are the shortage of qualified allergologists and of long-term clinical studies investigating allergic rhinitis as a life-long condition.

### Patient profiles, practical treatment guidelines, and associated challenges or limitations for urticaria

Sixty per cent of dermatologists and allergologists described acute urticaria as being the ones most commonly reported amongst their patients. The survey results also reveal the common patient profiles, unmet needs, key decision points for treatment, and practical considerations and/or limitations in treatment of patients with urticaria (Tables 1, 2). Specifically, the most common patient profiles are 20‒40-year-old adults, often without concomitant disease, and pregnant/lactating women.

The pattern of symptoms of chronic urticaria (for example, intermittent or continuous wheals, and characteristic signs on wheals and flares that resolve within 24 h without residual hyperpigmentation), clinical history, and trigger factors influence the diagnosis of urticaria and therefore its treatment. Table 3 describes the criteria for defining responders versus nonresponders to treatment, and controlled versus uncontrolled symptoms in the context of urticaria based on the weekly urticaria activity score, and the change in the number and frequency of urticaria lesions. However, 40% of dermatologists and allergologists still highlighted the requirement for additional tools for the diagnosis of specific subtypes of urticaria and a need to further disseminate and implement EAACI/GA(2)LEN/EDF/WAO guidelines.

Results show that the choice of treatment is dependent on the onset of the drug (preferably with extremely fast onset), drug potency (preferably high), and dosing regimen (preferably once-daily use). The patient’s desire to obtain treatment following diagnosis is also important in determining the course of treatment. In addition, other factors that influence treatment decision include patient age, response to treatment, disease severity, and affordability. Furthermore, caution should be employed when prescribing antihistamines to patients with renal or hepatic impairment, and elderly patients should not take sedating antihistamines. According to 60% of of dermatologists and allergologists surveyed in this study, patients should also have no adverse effects (e.g. central nervous system, cardiac) to the drugs prescribed and treatment should preferably not cause sedation. Other factors that may influence treatment decisions include the level of distress experienced due to the condition, the patient’s occupation and pregnancy status, and previous experience with antihistamines.

High drug costs and limited drug availability are some of the challenges identified by 40% of dermatologists and allergologists for the implementation of existing treatment guidelines for urticaria. Financial constrains often pose a challenge, especially in poor Asian countries where patients are unable to afford a regular dose or increased dose of a drug. Furthermore, institutional practices limiting drug prescriptions to specific agents also pose a challenge to the implementation of these guidelines.

In addition, the survey reiterated that patient adherence has an impact on the management of urticaria. Patients are often hesitant to continue treatment should they not experience immediate relief, despite advice that continued treatment with antihistamines for at least 2 weeks would be required for symptom improvements. The need for a completely non-sedating antihistamine and one that would restore the patient’s quality of life was thus highlighted to enhance patient treatment experience, particularly if they have jobs requiring high concentration. The high expense of some antihistamines and drugs such as omalizumab, specialist reluctance to change management styles, and doctor or patient preference for sedating antihistamines owing to a lack of knowledge on the sedative effects of first-generation antihistamines, limit the usefulness and practical applicability of current treatment guidelines.

Apart from these challenges, 20% of dermatologists and allergologists indicated that many patients are still embarrassed by the itch and rash, impacting their quality of life. Therefore, it was highlighted that better treatment strategies for control of symptoms amongst urticaria patients, their symptoms often leading to lack of/disturbance of sleep, are required. A need for more suitable drugs for those with renal impairment was also cited by 20% of these clinicians.

### Selection of second-generation antihistamines in the treatment of allergic rhinitis and urticaria

Many considerations come into play when selecting the optimal antihistamine for patients. Ninety per cent of clinicians surveyed cited drug efficacy and safety as the most important consideration. This is followed by drug impact on sedation and psychomotor function (60%), patient lifestyle (50%) and treatment adherence (50%) (Table 4). Other considerations include concomitant disease, cost, pregnancy and breastfeeding, presence of renal or hepatic problems, cardiac disease and side effects, and consumption of other medications.

**Table 4:** Important factors when selecting the most suitable second-generation antihistamine

	Most important			Least important	
Efficacy and safety	9	1			
Lack of sedation	6	4			
Lack of psychomotor impairment	6	3	1		
Adherence to treatment	5	2	2	1	
Lifestyle of patients (e.g., level of daily activity, type of employment)	5	1	3		1
Concomitant disease	4		2		2
Costs of treatment	3	3	3		1
Special patient populations	3	4	2	1	
Others: lack of cardiac side effects	1				
Others: previous antihistamines used	1				

Numbers in the cells indicate the number of responses received from clinicians for each of these criteria

This survey highlighted that patient lifestyle also influences the selection of antihistamine; sixty per cent of clinicians stated a need to prescribe non-sedating antihistamines for patients who need to stay alert and require excellent psychomotor responses, and those who are active in sports or outdoor activities. Of note was the need to consider patient adherence/compliance as part of treatment decision-making in the management of not just of allergic rhinitis but—importantly—urticaria. Patients generally prefer to take antihistamines once daily, and those with difficulty adhering to treatment could be prescribed longer-acting, non-sedating drugs such as bilastine and fexofenadine [16, 18]. A good balance between drug efficacy and safety, with no cardiac or central nervous system adverse effects or drug interactions, should be considered in selecting the optimal antihistamine. In this study, the consensus among the surveyed clinicians was that bilastine was the first-choice antihistamine for both allergic rhinitis and urticaria, followed by fexofenadine.

Patients must also be clearly willing and able to pay for the antihistamines prescribed. Otherwise, cheaper alternatives need to be offered to these patients.

**Fig. 1:**
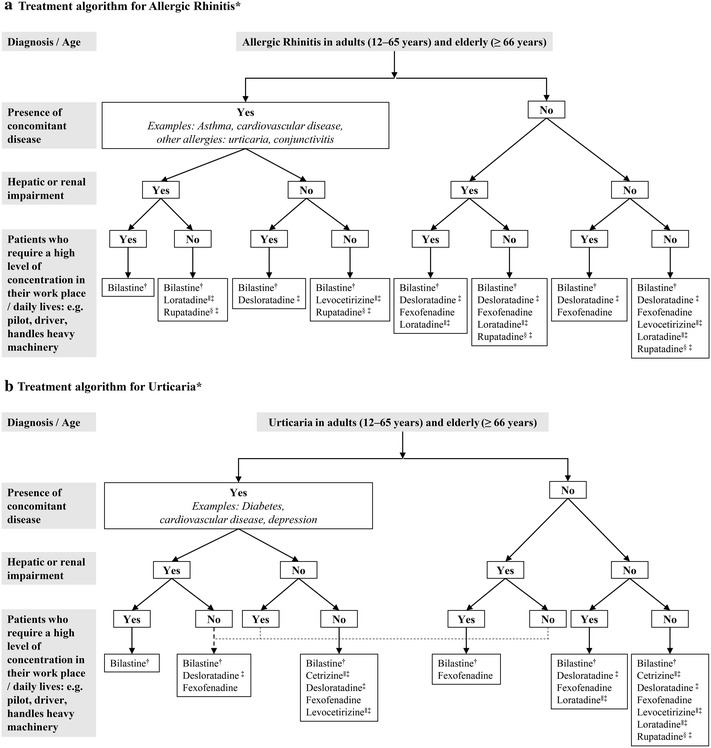
Algorithms for selecting second-generation antihistamines for allergic rhinitis and urticaria based on patient profiles. The choice of drugs has been listed in alphabetical order, not by preference. *Based on ARIA and EAACI/GA(2)LEN/EDF/WAO guidelines for allergic rhinitis and urticaria [6, 19]. ^†^Preferred antihistamine in patients with cardiac problems or those who are likely to consume alcohol. ^‡^Caution should be observed when prescribing antihistamines for elderly patients. ^§^Availability in Asia–Pacific countries is limited. ^∥^Pregnancy Category B (should be used in pregnancy if clearly needed)

Considering the factors listed above, an algorithm to triage patients suffering from allergic rhinitis and urticaria to a selection of the optimal antihistamines is suggested in this manuscript based on the ARIA and EAACI/GA(2)LEN/EDF/WAO guidelines for allergic rhinitis and urticaria [6, 19] (Fig. 1a, b). The most versatile antihistamines for adults and the elderly, as shown in Fig. 1, are bilastine and fexofenadine, bearing in mind patients who might already be taking other drugs (drug-to-drug interactions) and suitability for patients with renal and/or hepatic impairment. As second-generation antihistamines are non-sedative, they are all suitable for patients who lead an active lifestyle (Fig. 1). Consensus among the clinicians indicated a preference in prescribing bilastine or fexofenadine to patients who may have had prior experience with other second-generation antihistamines. Further, the survey revealed that bilastine was the preferred antihistamine of choice for all the patient groups discussed here, i.e., patients with either allergic rhinitis or urticaria, those with or without concomitant disease, hepatic or renal impairment, or those requiring a high level of concentration at work or in their daily lives (Fig. 1).

Clinicians surveyed were also asked to indicate their preferences and collective experiences for the treatment of allergic rhinitis and urticaria using the various second-generation antihistamines. While all second-generation antihistamines listed in Table 5 have been indicated for the treatment of both allergic rhinitis and urticaria, with the exceptions of bilastine, cetirizine, levocetirizine, and rupatadine, the remainder are not indicated for the treatment of allergic rhinoconjunctivitis (Table 5). Clinicians also indicated that due to their earlier approval and availability, the most frequently prescribed drugs for allergic rhinitis and urticaria are cetirizine and levocetirizine. However, with the greater availability of more novel second-generation antihistamines such as bilastine, along with comparable efficacy and greater safety, prescription patterns have started to change, particularly for patients described in Fig. 1. They also indicated that desloratadine works well, especially for patients with chronic urticaria. Table 5 summarizes the observations on efficacy, the commonly prescribed daily dose, and duration of treatment, dose adjustments required and contraindications/adverse effects associated with these antihistamines. Treatment duration for allergic rhinitis and urticaria using second-generation antihistamines ranged from 2 weeks to more than a year, depending on the severity of symptoms. All the antihistamines listed in Table 5 are available in the following APAC countries: Hong Kong, the Philippines, Singapore, and Thailand; bilastine is not yet available in Vietnam, and rupatadine is not available in Malaysia and was only recently introduced in the Philippines and Thailand.

**Table 5:** Clinical profile differences and second-generation antihistamine usage based on collective experiences of clinicians surveyed

	Bilastine	Cetirizine	Desloratadine	Fexofenadine	Levocetirizine	Loratadine	Rupatadine^a^
Indicated for:							
Allergic rhino-conjunctivitis	✓	✓	X	X	✓	X	✓
Urticaria	✓	✓	✓	✓	✓	✓	✓
Allergic rhinitis	✓	✓	✓	✓	✓	✓	✓
Most frequently prescribed for allergic rhinitis	Cetirizine						
Most frequently prescribed for urticaria	Cetirizine Levocetirizine						
Observations on efficacy	Excellent	Good	Fair/good	Excellent	Excellent	Good	Good
Commonly prescribed daily dose	20 mg OD	10 mg OD	5 mg OD	180 mg OD	5 mg OD	10 mg OD	10 mg OD
Duration of treatment	2 weeks and above (depending on severity of symptoms)						
Dose adjustment requirements	None	Severe renal impairment	Severe renal impairment	None	Severe renal impairment	Severe hepatic impairment	None
Contraindications and adverse effects	None	Severe renal impairment Sedation	None Light sedation	None Very occasional worsening (could be due to allergy to dye of pill)	Severe renal impairment Light sedation	None Sedation	Interaction with ketoconazole, erythromycin, and statins

Data based on United Kingdom SmPCs and/or clinician survey. In some APAC countries, only certain formulations of cetirizine are approved for allergic rhinoconjunctivitis

*OD* once daily

^a^Rupatadine is not available in Malaysia and was only recently introduced into the Philippines and Thailand; hence, experience using this drug may be limited

Dose adjustments were typically done with increments in the daily dose of up to four-fold for urticaria if there was no improvement of symptoms between 1 and 4 weeks. Additionally, second-generation antihistamines may be switched if patients experienced side effects, due to nonavailability of the drug or upon the patient’s request for a cheaper alternative. In the context of urticaria, using combinations of antihistamines is not usually found to be particularly beneficial in alleviating the patient’s symptoms. Meanwhile, for allergic rhinitis, some clinicians would opt to change the patient’s antihistamine if the patient reports unsatisfactory symptoms after 4‒6 weeks of continuous treatment. Others would change the second-generation antihistamine if there is not at least a 50% improvement in symptoms following 1 week of treatment, or if the patient complains of side effects other than sedation, such as chest pains, dizziness, dry mouth, or palpitations. Additionally, nasal endoscopy may be performed to eliminate the possibility of nasal polyps, sinusitis or deviated nasal septum. If treatment is not effective with a second-generation antihistamine alone, it is often combined with a nasal steroid spray. A skin prick test and/or nasal allergen provocation test may also be necessary at this point (Some of the survey results included off-label use of antihistamine preparations or combinations, which are not discussed in this manuscript).

Seventy per cent of clinicians highlighted that the benefits of second-generation antihistamines were their non-sedating properties, once-daily dosing and quick onset of action compared with nasal sprays. In addition, many of these newer antihistamines led to fewer anticholinergic effects and drug-to-drug interactions, longer duration of action and anti-inflammatory properties when dosage was increased. Nonetheless, drug costs and safety of some of these antihistamines for the hepatic/renal-impaired patients and those taking other drugs was a major consideration.

Of note were the beneficial properties and characteristics of the second-generation antihistamines bilastine and fexofenadine, as specified by all clinicians: non-sedative, once-daily dosing, highly efficacious, lack of cardiac side effects and safe even for the elderly. Additionally, dose adjustments are not required for patient with hepatic or renal impairment and no severe drug-to-drug interactions have been reported.

## Discussion

In the survey, the perceived adherence by specialists of GPs to the ARIA guidelines were high, at 70%, compared with the much lower adherence to the EAACI/GA(2)LEN/EDF/WAO guidelines (27%). The reason for there being such a large disparity between these estimates remains to be determined. Some GPs also choose to treat patients with first-generation antihistamines. An international multicenter study demonstrated that 82.5% (n = 193) of physicians were aware of the ARIA guidelines; overall, 84.2% (n = 197) and 84.6% (n = 198) of physicians felt that the guidelines were useful for categorizing patients and determining the best treatment, respectively [8]. Meanwhile, a study in Italy showed that allergic rhinitis is largely diagnosed by GPs (68%), and that awareness of ARIA guidelines is low [20].

These results demonstrate the unmet need and necessity to encourage more GPs and clinicians to use second-generation antihistamines as the first-line treatment for allergic rhinitis and urticaria due to their good efficacy and tolerability profiles, as recommended by the ARIA and EAACI/GA(2)LEN/EDF/WAO guidelines. It should also be noted that this study details the opinions of experts in the APAC region and does not account for assumptions related to the efficacy or safety of specific antihistamines over the others. However, results from this study and published literature highlight many challenges that lie ahead for the implementation of existing treatment guidelines, and increasing clinician and patient awareness of the existence of these guidelines could improve the existing clinical practices.

Second-generation antihistamines have been the standard of care and first-line treatment for allergic rhinitis and urticaria according to the ARIA and EAACI/GA(2)LEN/EDF/WAO guidelines [6, 19]. However, the factors highlighted in this study limit the use of some second-generation antihistamines due to fears of drug-to-drug interactions. In renal- or hepatic-impaired patients, dosages of these antihistamines may occasionally have to be adjusted. Nevertheless, the availability of newer second-generation antihistamines has generated interest in recent years in evaluating these newer compounds for their efficacy and safety, to assess if they meet the optimal profile for the management of allergic rhinitis and urticaria [18, 21–23].

The finding that bilastine, with its positive properties and characteristics, was the first-choice antihistamine amongst the surveyed clinicians, despite the need to take it on an empty stomach (noted as a minor inconvenience for patients), agrees with a recent review by Wang et al. indicating that this compound “has the highest number of desired features for a modern antihistamine according to international ARIA guidelines” for the management of allergic rhinitis when compared with other second-generation antihistamines [21]. Church and Lageaga [22] also further support the current survey outcome, noting that bilastine is an “ideal antihistamine for updosing in difficult-to-treat urticaria as recommended by the EAACI/GALEN/EDF/WAO guidelines for the management of urticaria”. Fexofenadine is also a preferred antihistamine as aligned with both ARIA and EAACI/GALEN/EDF/WAO guidelines [6, 19], although alternatives might prove more affordable for patients. Moreover, it is also important to consider the level of cost-effectiveness these cheaper alternatives could provide, compared with second-generation antihistamines, particularly in the perspective of efficacy and quality of life. Communication is thus critical in treatment decision-making, to help patients understand their options and ultimately the medications they will be receiving.

All second-generation antihistamines have been shown to be non-sedative; however, of these, bilastine, the newest-marketed non-sedating antihistamine, has been shown to have one of the lowest cerebral histamine H1 receptor occupancies compared with other modern second-generation antihistamines [24, 25]. Recent studies have also demonstrated that the benefit-to-risk ratio of bilastine is optimal, and that it is shown to be non-sedating and meets conditions for safety in drivers requiring antihistamines [25].

The use of some antihistamines is confined to selected countries. In countries like the Philippines and Thailand, generic antihistamines are available cheaply; however, there is a concern with respect to drug quality, resulting in the preference for the use of more affordable innovator drugs such as bilastine. In Thailand, however, clinicians indicated that their experience with newer antihistamines such as bilastine is minimal, due to limited availability. Additionally, the drug pricing differs between countries, and is highly dependent on the healthcare structure. A cost-effectiveness study comparing all the second-generation antihistamines will be useful in the APAC setting. Country-specific treatment preferences also come into play in drug selection; in the treatment of chronic spontaneous urticaria, Malaysian dermatologists may consider changing the type of second-generation antihistamine at least once if the patient does not respond to increments of dosage up to four-fold, before adding on other drugs. Examples of these include montelukast, cyclosporine, or omalizumab. Hence, drug prices and country-specific recommendations may also govern the selection of antihistamines for allergic rhinitis and urticaria treatment.

## Conclusions

The current manuscript provides the views of dermatologists, ENT specialists, and allergologists in the APAC region on the current ARIA and EAACI/GA(2)LEN/EDF/WAO guidelines and the practical considerations to improve the standard of patient care in APAC. Compliance with the ARIA and EAACI/GA(2)LEN/EDF/WAO guidelines is generally present in the region among dermatologists, allergologists and ENT specialists; however, it is lower amongst GPs. It is therefore important to conduct allergy education programs targeted at GPs as well as at patients. Updates to the existing guidelines may be useful in APAC countries to reflect the different patient profiles and varying symptoms of allergic rhinitis and urticaria observed. Triaging of patients based on various patients’ profiles, such as that proposed in this manuscript, would support clinicians in providing their patients with the best treatment options for their condition.

Nonetheless, all clinicians agreed that, currently, second-generation antihistamines should be used as the first line of treatment. There are a number of second-generation antihistamines that have come to the market in recent times, giving clinicians the opportunity to prescribe the best treatment, both to address the clinical symptoms and maximally improve their patients’ quality of life. Of note is the availability of second-generation antihistamines such as bilastine and fexofenadine, which are able to provide non-sedative benefits alongside being efficacious and safe. The APAC experts in this study, however, chose bilastine as the preferred choice of second-generation antihistamine due to its efficacy and tolerability, and its potential to be offered as a more cost-effective treatment for Asian patients.

## Additional file

**Additional file 1.** The supplementary material contains the survey that was put forth to the clinical experts as part of the study design.

## Abbreviations

APAC: Asia Pacific
ARIA: Allergic Rhinitis and its Impact on Asthma
EAACI: European Academy of Allergology and Clinical Immunology
EDF: European Dermatology Forum
ENT: Ear, Nose, and Throat
GP: General practitioner
GA(2)LEN: Global Allergy and Asthma European Network
WAO: World Allergy Organization
