# Supplementary material for: Selecting optimal second-generation antihistamines for allergic rhinitis and urticaria in Asia
Source: Clin Mol Allergy. 2017 Nov 1;15:19. doi: 10.1186/s12948-017-0074-3 (PMC5664819; doi:10.1186/s12948-017-0074-3)
Supplement: Supplementary file 1 — Additional file 1. The supplementary material contains the survey that was put forth to the clinical experts as part of the study design. [file 12948_2017_74_MOESM1_ESM.docx]

**Supplementary material**

Section 1: General information

This section aims to gather information about your areas of practice, your awareness and adherence to existing guidelines used in the treatment of persistent Allergic Rhinitis and Urticaria

1. Please state your name: __________________________
2. Please state your country of practice: _______________________
3. Please state your specialty: _____________________________
4. Which is the most common type of allergic disease that you see in your clinical practice?

| 1. Intermittent allergies |  |
| --- | --- |
| 1. Persistent allergies |  |
| 1. Seasonal allergies |  |
| 1. Others, please specify |  |

1. Are you aware and familiar with the existing ARIA and EAACI/GA2LEN/EDF/WAO guidelines for the diagnosis and treatment of Allergic Rhinitis and Urticaria respectively?

|  | Yes | No |
| --- | --- | --- |
| **ARIA** |  |  |
| **EAACI/GA2LEN/EDF/WAO** |  |  |

*Allergic Rhinitis and its Impact on Asthma (ARIA); European Academy of Allergology and Clinical Immunology (EAACI), the EU-funded network of excellence, the Global Allergy and Asthma European Network (GA2LEN), the European Dermatology Forum (EDF) and the World Allergy Organization (WAO).*

1. In your clinical practice, do you follow these guidelines?

|  | Yes | No | Partially |
| --- | --- | --- | --- |
| **ARIA** |  |  |  |
| **EAACI/GA2LEN/EDF/WAO** |  |  |  |
| **Other guidelines** |  |  |  |
| *Please specify:* | | | |

1. If you answered “Yes/ Partially” to the previous question, please share any practical considerations you may have about these clinical guidelines. If you have indicated “other guidelines”, please specify which. If you have answered “No/Partially”, kindly indicate the reasons for non-adherence.

| Yes or Partially |  |
| --- | --- |
| No or Partially |  |

1. In your observations in the clinical practice, do your peers follow these guidelines, irrespective of the severity of presenting symptoms?

|  | Yes | No | Partially |
| --- | --- | --- | --- |
| **ARIA** |  |  |  |
| **EAACI/GA2LEN/EDF/WAO** |  |  |  |
| **Other guidelines** |  |  |  |
| *Please specify:* | | | |

1. If you answered “No/ Partially” to the previous question, please share your thoughts on what prevents them from following the guidelines

| No or Partially |  |
| --- | --- |

1. In your experience, do your General Practitioners follow these guidelines, regardless of the severity of presenting symptoms? What percentage of GPs do you estimate follow the guidelines?

|  | Yes | No | Partially |
| --- | --- | --- | --- |
| **ARIA** | __________ % (estimated) | __________ % (estimated) | __________ % (estimated) |
| **EAACI/GA2LEN/EDF/WAO** | __________ % (estimated) | __________ % (estimated) | __________ % (estimated) |

1. If you answered “No/Partially” to the previous question, please share your thoughts on what prevents them from following the guidelines

| No or Partially |  |
| --- | --- |

*Please continue to section 2 on next page.*

Section 2: Patient profiles and practical treatment guidelines in your country of practice

This section aims to understand the different patient profiles for Allergic Rhinitis and Urticaria in your clinical practice and to understand the practical considerations and/or limitations in your experiences for determining the preferred method of diagnosis and treatment.

**Persistent Allergic Rhinitis (≥4 days per week and ≥4 weeks)**

1. Please describe the different patient profiles (e.g. gender, age groups, concomitant disease, special patient populations, lifestyle impacted by chronic disease) and associated symptoms (e.g. varying severity or combinations of symptoms, symptoms most frequently reported by patients before and during treatment) that you observe in your clinical practice.

Comment: _________________________________________

1. From your experience, are there any unmet needs in the diagnosis and treatment of patients with persistent Allergic Rhinitis?
   1. Yes , please comment:
   2. No
2. Considering the different patient profiles and the varying severity of symptoms of patients who present with Allergic Rhinitis in your country of practice, please describe the key decision points and propose recommendations for diagnosing and treating Allergic Rhinitis. Please include details about the following in your response:
   1. Patient profiles and associated symptoms for persistent allergic rhinitis:

- 1. Key decision points for diagnosis:
  2. Key decision points for treatment:
  3. The importance of patient preference when selecting an antihistamine for treatment:
  4. Any other considerations:

Please see the ARIA guidelines attached. You may choose to propose updates based on these guidelines or provide other recommendations covering items (a)-(d) above. Please indicated [*sic*] if recommendations are based on scientific evidence (please provide relevant references) or your personal clinical practice. If you choose to propose updates to the ARIA guidelines in the form of a modified flow chart, kindly send in your responses via email.

1. Do you see any challenges for the implementation of existing treatment guidelines for Allergic Rhinitis in the Asia Pacific region?
   1. Yes , please elaborate:
   2. No
2. What are the practical considerations and/or limitations in your experience that lead to your recommendations in Questions above? I.e. considerations related to your patient profiles, preferred method of diagnosis and treatment for persistent Allergic Rhinitis.
   1. Practical considerations:
   2. Limitations:

**Chronic Urticaria**

1. Please describe the different patient profiles (e.g. gender, age groups, concomitant disease, special patient populations, lifestyle impacted by chronic disease/quality of life) and associated symptoms (e.g. varying severity or combinations of symptoms, symptoms most frequently reported by patients before and during treatment) that you observe in your clinical practice.

Comment: _________________________________________

1. From your experience, are there any unmet needs in the diagnosis and treatment of patients with urticaria?
   1. Yes , please comment:
   2. No
2. Considering the different patient profiles and the varying severity of symptoms of patient who present with Urticaria in your country of practice, please describe the key decision points and propose recommendations for diagnosing and treating Urticaria. Please include details about the following in your response:
   1. Patient profiles and associated symptoms for persistent allergic rhinitis:

- 1. Key decision points for diagnosis:
  2. Key decision points for treatment:
  3. The importance of patient preference when selecting an antihistamine for treatment:
  4. Any other considerations:

Please see the EAACI/GA2LEN/EDF/WAO guidelines attached. You may choose to propose updates based on these guidelines or provide other recommendations covering items (a)-(d) above. Please indicate if recommendations are based on scientific evidence (please provide relevant references) or your personal clinical practice.

If you choose to propose updates to the EAACI/GA2LEN/EDF/WAO guidelines in the form of a modified flow chart, kindly send in your responses via email.

1. Do you see any challenges for the implementation of existing treatment guidelines for Allergic Rhinitis in the Asia Pacific region?
   1. Yes , please elaborate:
   2. No
2. What are the practical considerations and/or limitations in your experience that lead to your recommendations in Questions above? I.e. considerations related to your patient profiles, preferred method of diagnosis and treatment for chronic Urticaria.
   1. Practical considerations:
   2. Limitations:

*Please continue to section 3 on next page.*

Section 3: Treatment with second generation antihistamines

The ARIA and EAACI/GA2LEN/EDF/WAO guidelines recommend second generation antihistamines as the first-line of treatment for Allergic Rhinitis and Urticaria. This section aims to gather information about your preferences and considerations when treating patients with these conditions using second generation antihistamines

1. Second generation antihistamines are commonly used as the first line of treatment for Allergic Rhinitis and Urticaria. Please prioritize your considerations (1=most important to 5=least important) when selecting antihistamines for optimal treatment of your patients bearing in mind the following factors:

|  | 1 | 2 | 3 | 4 | 5 |
| --- | --- | --- | --- | --- | --- |
| Concomitant disease |  |  |  |  |  |
| Lifestyle of patients (e.g. level of daily activity, type of employment) |  |  |  |  |  |
| Costs of treatment |  |  |  |  |  |
| Special patient populations |  |  |  |  |  |
| Adherence to treatment |  |  |  |  |  |
| Efficacy and safety |  |  |  |  |  |
| Lack of sedation |  |  |  |  |  |
| Lack of psychomotor impairment |  |  |  |  |  |
| Others: |  |  |  |  |  |

1. Based on your responses from the question above, kindly provide details of your considerations for the following factors when selecting antihistamines for optimal treatment of your patients:
   1. Concomitant disease:
   2. Lifestyle of patients e.g. level of activity, nature of employment:
   3. Costs of treatment:
   4. Special patient populations:
   5. Adherence to treatment:
   6. Efficacy and safety:
   7. Others:
2. Please provide details of the antihistamine options currently available in your country of practice by completing the following table:

|  | **Bilastine** | **Cetirizine** | **Desloratadine** | **Fexofenadine** | **Levocetirizine** | **Loratadine** | **Others**: |
| --- | --- | --- | --- | --- | --- | --- | --- |
| Indicated for the treatment of allergic rhinoconjunctivitis | Yes/No | Yes/No | Yes/No | Yes/No | Yes/No | Yes/No | Yes/No |
| Indicated for the treatment of any type of urticaria | Yes/No | Yes/No | Yes/No | Yes/No | Yes/No | Yes/No | Yes/No |
| Most frequently prescribed antihistamine for allergic rhinitis *(please check where applicable)* |  |  |  |  |  |  |  |
| Most frequently prescribed antihistamine for urticaria *(please tick where applicable)* |  |  |  |  |  |  |  |
| Observations on efficacy |  |  |  |  |  |  |  |
| Conditions commonly treated with this antihistamine *(AR/ U/ both: please state)* | AR:  U:  Both: | AR:  U:  Both: | AR:  U:  Both: | AR:  U:  Both: | AR:  U:  Both: | AR:  U:  Both: | AR:  U:  Both: |
| Commonly prescribed daily dosage for adults *(please specify condition and severity of the condition)* |  |  |  |  |  |  |  |
| Dose adjustment required |  |  |  |  |  |  |  |
| Duration of treatment |  |  |  |  |  |  |  |
| Contraindications and Adverse Effects |  |  |  |  |  |  |  |

1. Please provide details of any clinical studies you are aware of that may influence your decision to choose one antihistamine over the other and reasons why (e.g. a particular antihistamine that has been tested in treatment of particular conditions but not others, efficacy and associated side effects). Kindly include the respective references as well.

Comment:

1. Are there any benefits or considerations of second generation antihistamines that should be considered in this manuscript?
   1. Benefits:
   2. Considerations:
2. What are your opinions on the benefits or considerations of bilastine compared to other available second generation antihistamines in the treatment of allergic rhinitis and urticaria?
   1. Benefits:
   2. Considerations:
